# Saccharin

**Published:** 1888-02

**Authors:** 


### SACCHARIN.

Among the most remarkable of all the marvelous products of the coal-tar industry, as Sir Henry Roscoe has observed, may be ranked the body to which the name of saccharin has been given. This new sweet compound was originally named anhydro-ortho-sulphamine benzoic acid—a ponderous appellation truly, and one to be avoided for trade purposes at any rate; but, at the same time, we do not think the name saccharin, although in some respects appropriate, should have been applied to this body, as the same term has been in use since 1880, to designate the carbohydrate originally discovered by Péligot amongst the products of the action of lime on glucose or levulose.

There is no doubt as to the great value of the discovery of this coal-tar toluene product; but while not grudging Dr. Fahlberg the well-merited reward of his arduous labors and his brilliant discovery, still we think the statement that saccharin was discovered by him needs some modification; for, according to Remsen, the able professor of chemistry in the Johns Hopkins University, this substance came to light in the course of an investigation undertaken and carried on by Fahlberg, under his (Professor Remsen's) direction and superintendence. The new body in question was, in fact, first described in their conjoint paper in Vol. XII of the *Berichte d. Deut. Chem. Gesellschaft*.

The manufacture of this saccharin, or benzoic sulfinide as it is termed by Remsen, is now effected on a large scale, and a plentiful supply of the toluene from which it is prepared is always to be had. This toluene, or methyl benzine ($C_6H_5CH_3$) is obtained in the destructive distillation of coal, as in gas manufacture, being usually prepared by the fractional distillation of that portion of the coal-tar oil boiling at 100°–120°C. This new product is really neither an acid nor an anhydride, although, when an aqueous solution is neutralized with metallic carbonates, salts are obtained. Salts are also formed with the alkalies, which are very soluble, crystallizing in small needles, and having a sweet taste. When fused with potash it is easily converted into salicylic acid.

Saccharin forms a white crystalline powder, which fuses at a temperature of 200°C., when it undergoes partial decomposition, giving off a characteristic odor; it is but slightly soluble in cold water, somewhat more so in warm water, its solubility being, however, greatly increased by the presence of alkalies or their carbonates. Alcohol also furnishes a good solvent for saccharin, particularly 80 per cent. solutions, and it is likewise freely soluble in warm glycerine; but its most characteristic property is certainly its sweetening power, possessing nearly three hundred times that of cane sugar, for while a solution of cane sugar loses its sweetness when diluted to 1 in 250, a sweet taste is still perceptible in a watery solution of saccharin containing 1 in 70,000.

As sugar is a carbo-hydrate, its solutions are liable to undergo fermentation; but as saccharin is an aromatic derivation, it is not liable to undergo this decomposition, and hence it may be freely employed without the fear of destructive fermentation occurring. But, besides this, as saccharin possesses considerable antiseptic powers, its use in medicine may be attended with advantage—as, for example, in cases of alkaline fermentation of urine, upon which it has been shown to exert a strongly retarding influence.

Stutzer, Aducco, and Mosso, from their experiments with this body, agree in the belief that it is innocent, so far as the organism is concerned; and Stutzer's experiments further show that its presence has no retarding influence on the digestion of either proteids or hydrocarbons.

However administered, saccharin soon finds its way into the urine unaltered, this fluid not being altered thereby. And, while producing no injurious effect on the system, it is found to agree both with invalids and healthy individuals, and no anxiety as to its effect upon the health need be felt, even when taken for lengthened periods. As much as thirty to eighty grains have been administered daily without producing any injurious effect, even upon the appetite. Saccharin may therefore be said to be neither a food nor a poison; but from its peculiar properties it may often be employed therapeutically with advantage. Thus, it may with benefit wholly or partially replace sugar in the diet where the latter is contra-indicated, as in diabetes, general obesity, torpid liver, marked gouty diathesis, chronic glandular diseases of children, and in certain senile affections in which various cystic and genito-urinary disorders occur. In two cases of diabetes in which we tried it we found its use attended with marked benefit.

From half a grain to a grain and a half of saccharin will sweeten a cup of tea or coffee. Small saccharin tablets, indeed, have been prepared for this purpose, and these are now being supplied, it is said, to the German army; for in a very small bottle of scarcely appreciable weight a soldier can carry enough saccharin for a week's supply.

There is a wide field for the application of saccharin to the production of sweetmeats and preserves, and in disguising the taste of bitter and nauseous preparations, as well as in preserving the properties of others. But it must always be carefully borne in mind that saccharin is to be regarded as a condiment, and not as a food like sugar, and can therefore replace sugar only in exceptional cases.

